# Symptom Clusters and Related Factors of Late Toxicities in Head and Neck Cancer Survivors After Radiation Therapy: A Cross-Sectional Study

**DOI:** 10.3390/nursrep16030103

**Published:** 2026-03-23

**Authors:** Tomoharu Genka, Midori Kamizato

**Affiliations:** Faculty of Nursing, Okinawa Prefectural College of Nursing, Naha-City 902-8513, Okinawa, Japan; kamizato@okinawa-nurs.ac.jp

**Keywords:** head and neck cancer, late toxicity, symptom cluster, cancer survivors, radiotherapy

## Abstract

**Background/Objectives**: Head and neck cancer survivors experience many late toxicities following radiation therapy. This study aims to identify symptom clusters of late toxicities and their related factors in head and neck cancer survivors. **Methods**: A cross-sectional study was conducted with 83 survivors (pharyngeal or laryngeal cancer) who had received radiation therapy at least one year earlier. Nine late toxicities were assessed using the Japanese version of the Patient Reported Outcomes version of the Common Terminology Criteria for Adverse Events (PRO-CTCAE) and a custom questionnaire. Quality of life (QoL) and related factors were evaluated with the European Organization for Research and Treatment of Cancer (EORTC QLQ-C 30), Hospital Anxiety and Depression Scale (HADS), UCLA Loneliness Scale, and Liebowitz Social Anxiety Scale (LSAS). Exploratory factor analyses and multiple regression analyses were performed. **Results**: All participants reported at least one symptom. Dry mouth (90.4%) and difficulty swallowing (72.3%) were particularly prevalent. Exploratory factor analysis (EFA) identified two symptom clusters (SCs): an oropharyngeal dysfunction cluster (pain, trismus, taste changes, difficulty swallowing, hoarseness) and a dry mouth cluster (dry mouth, sticky saliva). Regression analysis indicated that higher scores in both clusters were significantly associated with lower global QoL (oropharyngeal dysfunction SC: β = −0.427, *p* < 0.001; dry mouth SC: β = −0.268, *p* = 0.009). Chemoradiotherapy (CRT) was also significantly associated with higher cluster scores (oropharyngeal dysfunction SC: β = 0.233, *p* = 0.020; dry mouth SC: β = 0.343, *p* = 0.001). **Conclusions**: Late toxicities following radiation therapy include two clusters: oropharyngeal dysfunction cluster and dry mouth cluster. Head and neck cancer survivors with higher SC scores had lower global QoL scores and had undergone CRT. These findings may aid in the assessment and self-management support of head and neck cancer survivors after radiation therapy.

## 1. Introduction

Head and neck cancer is a general term for cancers that develop in the oral cavity, pharynx, hypopharynx, and larynx, with approximately 890,000 cases occurring worldwide each year [1]. In Japan, approximately 13,000 people are newly diagnosed with head and neck cancer annually [2]. Eighty percent of patients with head and neck cancer undergo radiation therapy, which plays an important role in preserving function [3]. However, radiation therapy can cause both acute and late toxicities. Acute toxicities during radiation therapy can hinder the completion of treatment; however, they tend to improve within a few months after the completion of treatment. In contrast, many of the late toxicities that appear over several years after radiation therapy are irreversible, progressively worsen over time, and negatively impact the patient’s life throughout the rest of their life [4,5].

Late toxicities following radiation therapy arise due to the microvascular and stromal connective tissue reactions induced by radiation, followed by irreversible fibrosis [4,6]. Specifically, these include dry mouth [7], difficulty swallowing [8], trismus [9], and taste changes [10]. The diversity of late toxicities that occur in a single type of cancer is a characteristic of head and neck cancer. The various symptoms make it difficult to manage these conditions [11].

Symptom clusters (SCs) are a concept that can be applied to the symptom management of patients with cancer who experience various symptoms. A SC is defined as a group of two or more symptoms that are interrelated and occur simultaneously [12]. Viewing symptoms as a cluster allows us to comprehensively and systematically assess symptoms and address multiple symptoms using a single intervention approach [13]. Therefore, viewing late toxicities as SCs may be useful for effectively managing these symptoms.

Mathew et al. conducted a systematic review of SCs in patients with head and neck cancer during and up to three months after radiation therapy [14], identifying two major SC concepts. However, most studies included in the review focused on patients during or immediately after radiation therapy rather than on long-term survivors experiencing late toxicities. While both acute and late toxicities share similar symptoms, the mechanisms and progression of these symptoms differ [6], suggesting that they have different symptom experiences and require different management strategies. However, studies specifically examining clusters of late toxicities occurring more than one year after treatment completion and their associated factors remain limited.

Therefore, this study focuses on the late toxicities experienced by head and neck cancer survivors, identifying the SCs and their related factors. This study may clarify symptom experiences among head and neck cancer survivors undergoing radiation therapy and contribute to symptom assessment and self-management.

## 2. Materials and Methods

This cross-sectional observational study aimed to identify symptom clusters of radiation-induced late toxicities in head and neck cancer survivors and to examine their related factors. The study was designed and reported in accordance with the Strengthening the Reporting of Observational Studies in Epidemiology (STROBE) guidelines for cross-sectional studies.

### 2.1. Participants

The subjects of this study were head and neck cancer survivors undergoing follow-up treatment at otolaryngology outpatient wards in four general hospitals in Okinawa, Japan, between September 2020 and April 2021.

The selection criteria included individuals diagnosed with pharyngeal or laryngeal cancer who had undergone radiation therapy more than one year ago to account for the effects of acute toxicity. This timeframe was selected based on our previously published literature review [15] and to minimize potential overlap with resolving acute toxicities. Additionally, subjects were required to be 20 years or older, to have completed curative radiation therapy, to consent to participate in the study, and to be capable of verbal communication in Japanese. No specific criteria were set regarding disease stage or radiation dose. Exclusion criteria included those who had undergone laryngectomy, or had been diagnosed with dementia. Patients who received additional cancer-related systemic therapy during the study period were excluded to avoid confounding effects on symptom severity.

### 2.2. Sample Size

The required sample size was calculated based on the recommendation that it should be five to 10 times the number of variables [16]. This study analyzed nine symptoms, necessitating approximately 45 to 90 subjects. This sample size was considered feasible for estimating the situation in the survey region, as approximately 150 individuals are diagnosed with pharyngeal or laryngeal cancer annually in Okinawa, Japan [17].

The sample size calculation was based on the initially specified nine symptoms included in the planned exploratory EFA. The exclusion of two symptoms (hearing loss and dental caries) occurred after factor extraction based on the predefined loading threshold and did not alter the original a priori sample size determination.

### 2.3. Study Instruments

In addition to basic attributes, this study examined the severity of late toxicities and related factors such as anxiety and depression, social anxiety, social isolation, and quality of life (QoL).

#### 2.3.1. Basic Attributes and Medical Characteristics

Regarding basic attributes, subjects were asked about their age, gender, height, weight at the time of the study, pre-radiation therapy weight, smoking and drinking habits, marital status, presence of cohabiting family members, and employment status. Questions on medical characteristics covered the diagnosis, treatment method, categorized as radiation therapy alone or concurrent chemoradiotherapy (CRT), radiation dose, irradiation method, time since radiation therapy, and surgical history.

#### 2.3.2. Late Toxicities

Late toxicities were assessed using the Japanese version of the Patient-Reported Outcomes version of the Common Terminology Criteria for Adverse Events (PRO-CTCAE) [18] and custom-made questionnaire items. The nine symptoms investigated as late toxicities were dry mouth [8], difficulty swallowing [19], mouth/throat sores [20], hoarseness [21], taste changes [10], sticky saliva [8], trismus [9], hearing loss [22], and dental caries [23]. Because no single validated Japanese language instrument comprehensively captures radiation-induced late toxicities in long term head and neck cancer survivors, PRO-CTCAE items were supplemented with additional clinically relevant items to ensure comprehensive symptom assessment.

The PRO-CTCAE was developed by Basch et al. and assesses the severity and frequency of 78 toxicities related to various types of cancer and treatments based on patient self-evaluation [24]. It allows for the selection of relevant adverse event questions according to the purpose of the study. In this study, five question items related to the severity of dry mouth, difficulty swallowing, mouth/throat sores, hoarseness, and taste changes were selected to assess symptom severity. Each symptom’s severity was rated on a five-point scale: “1: Not at all”, “2: Mild”, “3: Moderate”, “4: Severe”, and “5: Very severe”.

Additionally, four late toxicities not included in the PRO-CTCAE—sticky saliva, trismus, hearing loss, and dental caries—were assessed using custom-made questionnaire items. These symptoms were selected based on a previous study [15]. The specific wording of the items was developed with reference to other instruments used for patients with head and neck cancer [25,26], because no single validated Japanese language instrument comprehensively captured these clinically relevant late toxicities in long-term head and neck cancer survivors. Prior to the main survey, a pilot survey was conducted with head and neck cancer survivors, and the content validity of the items was discussed among the researchers. To maintain consistency in symptom assessment, these items were rated on the same five-point severity scale as the PRO-CTCAE.

#### 2.3.3. Anxiety and Depression

The degrees of anxiety and depression were assessed using the Japanese version of the Hospital Anxiety and Depression Scale (HADS) [27]. HADS, developed by Zigmond et al., measures anxiety and depression in patients with physical illnesses [28]. It comprises two subscales: depression and anxiety, each consisting of seven items. Each item is scored from 0 to 3, with higher scores indicating greater degrees of anxiety or depression. Clinical cut-off values are set at 8 or above for each subscale to indicate the presence of clinically significant anxiety or depression [29].

#### 2.3.4. Social Anxiety

Social anxiety was assessed using the Japanese version of the Liebowitz Social Anxiety Scale (LSAS-J) [30]. The LSAS-J, developed by Liebowitz, measures the severity of social anxiety [31]. The questionnaire comprises 24 items, evaluating the degree of fear and frequency of avoidance behavior in situations likely to provoke social anxiety on a 4-point scale from “0. Not at all” to “3. Very strongly feel/avoid (with probability of 2/3 or more or 100%)”. Higher scores indicate higher levels of social anxiety.

#### 2.3.5. Social Isolation

Social isolation was assessed using the Japanese version of the University of California Los Angeles Loneliness Scale, third edition (UCLA Loneliness Scale) [32]. The UCLA Loneliness Scale, developed by Russell et al., is a self-reported measure of loneliness [33]. The questionnaire comprises 20 items evaluating the frequency of experiences related to loneliness, with responses rated on a 4-point scale of “1. Never” to “4. Always”. Higher scores indicate greater degrees of loneliness.

#### 2.3.6. QoL

QoL was measured using the Japanese version of the European Organization for Research and Treatment of Cancer core questionnaire (EORTC QLQ-C30) [34]. Developed by Aaronson et al., this scale is designed for assessing the QoL of patients with cancer [35]. The EORTC QLQ-C30 comprises 30 items and evaluates global QoL, along with five functional scores (physical, role, cognitive, emotional, and social functioning) and common symptoms experienced by patients with cancer. This study used questions related to global QoL and the five functional scores (two items for global QoL, five items for physical functioning, two items for role functioning, two items for emotional functioning, two items for cognitive functioning, and two items for social functioning). Global QoL was rated on a 7-point scale from “1: Very poor” to “7: Very good”, while functional scores were rated on a 4-point scale from “1: Never” to “4: Very often”, with higher scores indicating better QoL and functional status.

### 2.4. Data Collection Method

Data were collected through anonymous self-administered questionnaires. Prior to recruitment, the researchers reviewed electronic medical records to obtain treatment-related information necessary for eligibility screening, including surgical history, receipt of chemotherapy, and time elapsed since completion of radiation therapy. When eligible participants visited the hospital, the researchers explained the purpose of the study and distributed the questionnaires to those who provided informed consent. Participants were asked to complete the questionnaires during outpatient wait times or after their appointments, and the completed questionnaires were collected. Information regarding radiation technique and total radiation dose was extracted from electronic medical records rather than obtained from participants to ensure accuracy.

Medical record data were extracted by a single investigator using standardized operational definitions and a predefined abstraction protocol. Because a single extractor performed the data abstraction, interrater reliability assessment was not applicable. Prior to analysis, data consistency and completeness were systematically verified as part of quality control procedures.

### 2.5. Analysis Method

The prevalence and severity of late toxicities were calculated based on the scores. Prevalence was determined by categorizing responses other than “1: Not at all” as positive for the symptom, and the number and percentage of affected individuals were computed. Severity was assessed by checking the normality of scores using the Kolmogorov–Smirnov test, and because normality was not confirmed, the median score for each symptom was calculated. Additionally, medians were calculated for anxiety, depression, social anxiety, social isolation, and QoL. Descriptive statistics were used for basic attributes.

Symptom cluster of late toxicities were identified using exploratory EFA on the late toxicity scores. The validity of the EFA was verified by calculating the Kaiser–Meyer–Olkin (KMO) measure of sampling adequacy and performing Bartlett’s test of sphericity. Principal axis factoring was used to extract factors, and factor rotation was conducted using promax rotation. Principal axis factoring was selected to identify latent constructs underlying correlated symptoms. A factor loading cut-off value of 0.40 was determined a priori based on previous symptom cluster studies [36] and methodological recommendations in EFA. Symptoms with factor loadings below this threshold on all factors were excluded from cluster construction. The number of factors was determined based on the Kaiser criterion (eigenvalues > 1). In exploratory SC research, the interpretability of the extracted factor structure was also considered. Furthermore, Cronbach’s α coefficient for each factor was calculated to assess internal consistency. For the identified SCs, the symptoms included were reviewed, and names for the SCs were assigned. The scores for symptoms within each cluster were summed to calculate the SC scores.

Associations of SC scores with related factors and QoL were first assessed using univariate analyses, including correlation coefficients, the Mann–Whitney U test, and the Kruskal–Wallis test, as appropriate. Subsequently, stepwise multiple regression analysis was conducted with SC scores as the dependent variable and the related factors, QoL scores, and basic attributes as the independent variables. Candidate independent variables were first screened using univariate analyses examining associations between SC scores and participant characteristics, related factors, and QoL scores. Variables showing statistically significant associations in these analyses were then entered into the stepwise regression model. Given the exploratory nature of this study and the balance between the sample size and the number of candidate variables, a stepwise selection procedure was used to screen for variables potentially associated with SC scores. Multicollinearity was assessed using variance inflation factors (VIF), and all values were 1.0. Treatment type was entered into the regression model as a dummy variable (1: radiation therapy alone, 2: CRT). The analysis was performed using IBM SPSS Statistics version 23, and statistical significance was set at *p* < 0.05.

## 3. Results

### 3.1. Sociodemographic and Clinical Features

The demographic and clinical characteristics of the participants are presented in Table 1. One hundred and fifty-three (153) individuals met the selection criteria during the study period. Of these, 85 consented to participate and received the questionnaire, and 83 returned completed questionnaires (response rate: 55.6%). The main reasons for those who could not cooperate with the study included “Lack of time and wanting to leave quickly” in 28 individuals; “Feeling unwell” in 20 individuals; “Lack of motivation” in eight individuals; “Fear of COVID-19” in four individuals; “Belief that they did not have any toxicity and were therefore not suitable for the study” in two individuals; “Called up for the medical examination while working on the questionnaire and went home right after” in two individuals; and “Other reasons” in four individuals.

The participants’ mean age was 64.4 ± 9.8 years (range 31–82), with most being male (67 participants, 80.7%). The most common diagnosis was oropharyngeal cancer in 32 participants (38.6%), and 70 participants (84.3%) had received CRT. The mean time since radiation therapy completion was 51.5 ± 34.2 months (range 12–179), with 15 participants (18.1%) at one year post treatment and 28 participants (33.7%) at five years or more after treatment. Among those who had surpassed five years, 22 participants were in the 5–10 year range and 6 were in the 10–20 year range, with a mean duration of 86.5 months.

### 3.2. Related Factors of Late Toxicities and QoL

The scores for related factors and QoL are presented in Table 2. The median anxiety score was 3.0, and the median depression score was 1.0. The number of participants exceeding the clinical cut-off values for anxiety and depression were 5 and 3 [27], respectively. The median scores for social isolation and social anxiety were 25.0 and 6.0, respectively. The median scores for QoL were 66.7 for global QoL and 93.3 for physical function QoL.

### 3.3. Prevalence and Severity of Late Toxicities

The prevalence and severity of late toxicities are presented in Table 3. All participants experienced at least one late toxicity, and among them, the highest prevalence was for dry mouth, affecting 75 participants (90.4%), followed by difficulty swallowing, affecting 60 participants (72.3%). Mouth/throat sores had the lowest prevalence among late toxicities; however, it still affected 20 participants (24.1%). The late toxicities with the highest severity scores were dry mouth and difficulty swallowing, each with a score of 3.0, followed by sticky saliva, hoarseness, and hearing loss with a severity score of 2.0.

### 3.4. SCs of Late Toxicities

The KMO value was 0.67, and Bartlett’s test of sphericity was significant (*p* < 0.001), confirming the appropriateness of the EFA [37]. The EFA extracted two factors. The number of factors was determined based on the Kaiser criterion (eigenvalues > 1). The eigenvalues of the retained factors were 3.17 for Factor 1 and 1.26 for Factor 2, accounting for 35.25% and 14.00% of the variance, respectively, with a cumulative contribution ratio of 49.25%. In the initial factor solution, mouth/throat sores, trismus, taste changes, difficulty swallowing, hoarseness, and hearing loss loaded on the first factor, while dry mouth, sticky saliva, and dental caries loaded on the second factor. Notably, hearing loss (Factor 1 = 0.226, Factor 2 = 0.214) and dental caries (Factor 1 = −0.183, Factor 2 = 0.209) were excluded because their factor loadings did not reach the predefined cut-off value of 0.40. After examining the relationships among late toxicities within each factor, the first factor was named the “Oropharyngeal dysfunction cluster” and the second factor the “Dry mouth cluster”. The results of the final EFA for late toxicities are shown in Table 4.

### 3.5. Association Between SC Scores and Basic Attributes

According to disease type, patients with oropharyngeal cancer had higher scores for both the oropharyngeal dysfunction and the dry mouth clusters compared to other groups (oropharyngeal dysfunction: *p* = 0.010; dry mouth: *p* = 0.030). Additionally, patients who received CRT had higher scores for both the oropharyngeal dysfunction and the dry mouth cluster (oropharyngeal dysfunction: *p* = 0.002; dry mouth: *p* = 0.020). There were no differences in SC scores based on the duration since the end of radiation treatment, smoking status, or alcohol consumption.

### 3.6. Correlation Between SC Scores, Related Factors and QoL

The correlation coefficients between SC scores, related factors, and QoL are presented in Table 5. These correlations were examined as an exploratory analysis to describe patterns of association between symptom clusters and related factors prior to multivariable modeling. The oropharyngeal dysfunction cluster score had significant positive correlations with anxiety (r = 0.36, *p* = 0.001), depression (r = 0.33, *p* = 0.002), social isolation (r = 0.21, *p* = 0.040), and social anxiety (r = 0.24, *p* = 0.030) and significant negative correlations with global QoL (r = −0.37, *p* = 0.001), physical functioning QoL (r = −0.35, *p* = 0.001), and role-functioning QoL (r = −0.24, *p* = 0.030). The dry mouth cluster had significant positive correlations with anxiety (r = 0.28, *p* = 0.010) and depression (r = 0.31, *p* = 0.004) and a significant negative correlation with global QoL (r = −0.26, *p* = 0.020). Furthermore, a significant positive correlation between the oropharyngeal dysfunction cluster and the dry mouth cluster (r = 0.44, *p* < 0.001) scores was observed.

### 3.7. Association Between SC Scores and Basic Attributes, Related Factors, and QoL

The associations between SC scores, basic attributes, related factors, and QoL are presented in Table 6. The multiple regression analysis indicated that higher scores in both the oropharyngeal dysfunction SC and the dry mouth cluster were significantly associated with lower global QoL scores (oropharyngeal dysfunction SC: β = −0.427, *p* < 0.001, dry mouth SC: β = −0.268, *p* = 0.009). In addition, CRT was significantly associated with higher scores for both the oropharyngeal dysfunction cluster and the dry mouth cluster (oropharyngeal dysfunction cluster: β = 0.233, *p* = 0.020; dry mouth cluster: β = 0.343, *p* = 0.001).

## 4. Discussion

This study investigated the SCs and related factors of late toxicities experienced by head and neck cancer survivors who had undergone radiation therapy over a year ago. The results revealed that the prevalence of dry mouth was highest among the late toxicities. Additionally, two SCs were identified from the nine late toxicities: the oropharyngeal dysfunction cluster (mouth/throat sores, trismus, taste changes, difficulty swallowing, and hoarseness) and the dry mouth cluster (dry mouth and sticky saliva). Furthermore, participants with high scores in both SCs had significantly lower global QoL scores and were more likely to have received CRT.

Previous studies on SCs in head and neck cancer have mainly examined acute or subacute toxicities [14]. During and up to three months after radiotherapy, symptoms such as dry mouth, mouth/throat sores, taste changes, and dysphagia have been grouped within a single oropharyngeal dysfunction cluster [14], likely reflecting inflammatory processes induced by radiation therapy [38]. In contrast, our findings suggest that in long-term survivorship, dry mouth and sticky saliva may form a distinct cluster separate from other oropharyngeal symptoms. This distinction underscores the importance of examining late toxicities specifically rather than extrapolating patterns from acute phase studies. The separation of xerostomia-related symptoms may characterize chronic survivorship after radiotherapy, where persistent salivary gland dysfunction becomes prominent [6].

The observed separation of clusters may reflect differences in underlying pathophysiological mechanisms. The oropharyngeal dysfunction cluster likely represents the combined effects of radiation-induced fibrosis and neuromuscular impairment affecting the oral and pharyngeal structures, contributing to persistent swallowing difficulty, trismus, hoarseness, and taste changes [39]. In contrast, the dry mouth cluster appears more closely related to irreversible salivary gland damage, with long term alterations in salivary secretion and composition resulting from radiation exposure [40]. These pathological processes differ in mechanism and temporal progression, suggesting they may not evolve in parallel over time and thus contribute to distinct symptom clustering patterns in the late phase of survivorship.

This study found that the scores for the dry mouth and oropharyngeal dysfunction SCs were correlated. Dry mouth has the highest prevalence among the late toxicities, and previous studies have reported that it can exacerbate other symptoms [41]. Therefore, symptoms in the dry mouth cluster and those in the oropharyngeal dysfunction cluster may influence each other, potentially worsening the symptom experience of head and neck cancer survivors. Thus, educating head and neck cancer survivors about management strategies for dry mouth as self-management support for them could potentially reduce symptoms in the oropharyngeal dysfunction cluster.

In this study, hearing loss and dental caries did not load strongly on either identified symptom cluster. Rather than suggesting these are unimportant, this pattern may reflect distinct mechanisms underlying these specific late toxicities in head and neck cancer survivors. Radiation caries is a well recognized complication after radiotherapy that develops over time as a result of multifactorial factors including salivary dysfunction, oral hygiene, and nutritional status in survivors [42]. Similarly, although hearing loss has been observed in head and neck cancer survivors [43], its prevalence and severity may depend on treatment specifics and individual risk factors that do not align directly with other late toxicity patterns and may evolve independently of clusters defined by neuromuscular or salivary dysfunction. Because these determinants can vary independently from the processes that underlie the clusters identified in our EFA, the absence of strong factor loadings for hearing loss and dental caries should be interpreted as reflecting multifactorial and partially independent late effects. This should not be taken to indicate reduced clinical importance.

This study found that individuals who had undergone CRT exhibited higher scores on both symptom clusters of late toxicities. Because CRT is typically selected for patients with more advanced disease or higher risk anatomy, this association should be interpreted cautiously and may partly reflect differences in baseline disease severity rather than a direct treatment effect. In addition, tumor stage, cumulative chemotherapy dose, and radiation dose to the salivary glands were not included in the present analysis, and these unmeasured clinical factors may have confounded the observed association. Nevertheless, biological plausibility exists. According to previous studies, CRT may be associated with the promotion of fibrosis and neuropathy [44,45]. Cancer treatment-related salivary dysfunction has also been described across modalities, including chemotherapy [46]. However, given the cross-sectional design, these findings should be interpreted as associative rather than causal.

### 4.1. Implications for Nursing

Our findings indicate that dry mouth may function as a practical indicator of the broader burden of late toxicities. Because many long-term survivors attend outpatient follow-up infrequently, brief screening for xerostomia using targeted questions about dryness and its functional impact may help identify individuals at risk for co-occurring swallowing or other oral functional impairments. Dry mouth is well recognized as a persistent late toxicities of head and neck radiotherapy and has been associated with functional limitations and reduced QoL [47]. Accordingly, incorporating a simple assessment of dry mouth into routine follow-up may facilitate the earlier identification of patients who require additional evaluation.

In addition, structured self-management education is particularly important in long-term survivorship. In Japan, support systems for cancer survivors more than 5 years after treatment remain insufficient, and opportunities for follow up may decrease over time. Therefore, during the period when patients are still attending regular follow up visits, nurses should support them in acquiring self-management skills. In this study, greater SC severity was associated with lower global quality of life. Prior evidence indicates that proactive symptom monitoring and patient education can facilitate earlier recognition of worsening late toxicities and contribute to improved functional outcomes [48,49]. Providing concise guidance on daily oral care, along with clear thresholds for early re-consultation (e.g., dehydration and malnutrition associated with dysphagia) may help prevent deterioration between annual visits, particularly among patients with limited access to specialized care. Furthermore, patients should be educated about the importance of seeking medical attention early if symptoms worsen or functional impairment becomes more severe after routine follow-up has ended, particularly beyond 5 years after treatment.

### 4.2. Limitations

This study has several limitations. The sample was restricted to patients with pharyngeal and laryngeal cancers treated with organ-preserving radiotherapy, and those who had undergone laryngectomy were excluded. Participants were recruited from outpatient clinics in a single geographical region, which may limit generalizability to other settings and survivorship populations. A relatively high refusal rate was observed, and detailed data for non-participants were unavailable; therefore, potential selection bias cannot be excluded. Accordingly, the study sample may underrepresent survivors with poorer health status, greater symptom burden.

The time since completion of radiotherapy varied widely among participants. Although additional analyses including time since treatment as a covariate did not materially alter the findings, the results of these additional analyses are presented in Supplementary Table S1. Temporal heterogeneity may have influenced cluster stability.

Methodologically, the sample size met commonly cited participant-to-variable ratios; however, it remained modest, and the KMO value indicated only moderate sampling adequacy. Parallel analysis was not performed, and the dry mouth cluster consisted of only two symptoms, with borderline internal consistency (Cronbach’s α = 0.68) potentially limiting psychometric robustness. The factor structure should therefore be considered exploratory and hypothesis-generating.

Four symptoms were assessed using custom items without formal psychometric validation, which may have affected measurement accuracy, but also the structural validity of the exploratory factor analysis.

Because the design was cross-sectional, temporal changes in SCs and the direction of associations with QoL cannot be determined. In addition, important clinical confounders, including tumor stage, cumulative chemotherapy dose, and radiation dose to the salivary glands, were not included in the analysis. The regression analyses were exploratory, and stepwise selection may increase the risk of model instability, inflated Type I error, sample dependency, and overfitting. In addition, cross validation was not performed; therefore, the identified associations may not be stable across other samples. Furthermore, the R^2^ values indicate that only part of the variance in SC severity was explained, suggesting that unmeasured biological, treatment-related, or psychosocial factors may also contribute.

## 5. Conclusions

Head and neck cancer survivors who had undergone radiation therapy over a year ago all exhibited at least one late toxicity, with dry mouth and difficulty swallowing being the most prevalent and severe. Furthermore, these late toxicities constituted two SCs: an oropharyngeal dysfunction cluster comprising mouth/throat sores, trismus, taste changes, difficulty swallowing, and hoarseness; and a dry mouth cluster comprising dry mouth and sticky saliva. Moreover, individuals with high scores in both SCs had lower global QoL scores and had received CRT. To our knowledge, this study is among the first to identify distinct SCs of late toxicities in head and neck cancer survivors after radiation therapy. This study’s findings suggest that the insights gained could be useful for assessing late toxicities following radiation therapy and for supporting self-management among head and neck cancer survivors.

## Figures and Tables

**Table 1:** Participants’ demographic and clinical characteristics (n = 83).

Characteristics		n (%)
Age (years)	<60	24 (29.0)
	60–69	28 (33.7)
	>70	31 (37.3)
Gender (%)	Male	67 (80.7)
	Female	16 (19.3)
BMI ^☆^	≤20.9	34 (41.0)
	21.0–27.9	46 (55.4)
	≥28.0	3 (3.6)
Smoking	Yes	14 (16.9)
	No	69 (83.1)
Alcohol consumption	Yes	40 (48.2)
	No	43 (51.8)
Marital status	Married	66 (79.5)
	Single ‡	17 (20.5)
On the job	Yes	45 (54.2)
	No	38 (45.8)
Tumor site	Nasopharynx	14 (16.9)
	Oropharynx	32 (38.6)
	Hypopharynx	16 (19.2)
	Larynx	21 (25.3)
Treatment type	Radiation alone	13 (15.7)
	CCRT	70 (84.3)
Survival years †	1	15 (18.1)
	2	16 (19.3)
	3	8 (9.6)
	4	16 (19.3)
	≥5 ‖	28 (33.7)
Radiotherapy Technique	IMRT	51 (61.4)
	non IMRT	32 (38.6)
Radiation dose (Gy)	>70	12 (14.5)
	≤70	71 (85.5)
Surgery	Yes	66 (79.5)
	No	17 (20.5)

^☆^ BMI: Body Mass Index (kg/m^2^). † Duration since completion of radiotherapy. Year 1: 12–23 months, Year 2: 24–34 months, Year 3–4: 36–48 months. ‡ Divorced and widowed included. ‖ 5 years or longer: 49 months or longer 5–10 years: 22 participants, 10–20 years: six participants, mean duration of 86.5 months. IMRT: Intensity Modulated Radiation Therapy.

**Table 2:** Psychosocial factors and quality of life (n = 83).

Related Factor	Score	Range
Anxiety ^☆^	3.0	(0–10)
Depression †	1.0	(0–10)
Social isolation ‡	25.0	(20–53)
Social anxiety §	6.0	(0–117)
Global quality of life ‖	66.7	(16.7–100)
Physical function ‖	93.3	(40.0–100)
Role function ‖	100.0	(33.3–100)
Emotional function ‖	83.3	(50.0–100)
Cognitive function ‖	83.3	(33.3–100)
Social function ‖	100.0	(33.3–100)

^☆^ HADS (Hospital Anxiety and Depression Scale)-Median anxiety score. † HADS (Hospital Anxiety and Depression Scale)-Median depression score. ‡ Median score for Japanese version of UCLA LS (Japanese version of University of California Los Angeles Loneliness Scale). § Median score for LSAS-J (Liebowitz Social Anxiety Scale). ‖ Median score for EORTCQLQ-C30 (European Organization for Research and Treatment of Cancer Quality of Life Questionnaire Core-30).

**Table 3:** Prevalence ^☆^ and severity † of late toxicities (n = 83).

Late Toxicities	n (%)	Severity (Range)
Dry mouth	75 (90.4)	3.0 (1–5)
Difficulty swallowing	60 (72.3)	3.0 (1–5)
Sticky saliva ‡	56 (67.5)	2.0 (1–5)
Hoarseness	54 (65.1)	2.0 (1–5)
Hearing loss ‡	43 (51.8)	2.0 (1–5)
Dental caries ‡	35 (42.2)	1.0 (1–5)
Trismus ‡	27 (32.5)	1.0 (1–5)
Taste changes	25 (30.1)	1.0 (1–5)
Mouth/throat sores	20 (24.1)	1.0 (1–5)

^☆^ Responses other than “1. Not at all” were considered positive for each symptom, and based hereon, the number of affected participants and prevalence were calculated. † Median score for each symptom (1: Not at all, 2: Mild, 3: Moderate, 4: Severe, 5: Very severe). ‡ Questions prepared by researchers.

**Table 4:** Factor analysis of late toxicities ^☆^ (n = 83).

Late Toxicities	Factor Loading		Cronbach’s Alpha
	Factor 1	Factor 2	
Mouth/Throat sores	0.902	−0.241	0.73
Trismus	0.639	−0.079	
Taste changes	0.564	0.191	
Difficulty swallowing	0.506	0.190	
Hoarseness	0.406	0.018	
Dry mouth	0.104	0.880	0.68
Sticky saliva	0.015	0.406	
Contribution ratio (%)	35.25	14.00	
Cumulative contribution ratio (%)	35.25	49.25	

^☆^ Hearing loss (factor loading: Factor 1 = 0.226, Factor 2 = 0.214) and dental caries (factor loading: Factor 1 = −0.183, Factor 2 = 0.209) were excluded from the late toxicities. Excluded items are reported in the footnote only.

**Table 5:** Correlation coefficients between SC scores and psychosocial factors and quality of life ^☆^.

Symptom Cluster		Anxiety	Depression	Social Isolation	Social Anxiety	Global	Physical	Role	Emotional	Cognitive	Social
Oropharyngeal dysfunction SC †	r	0.36	0.33	0.21	0.24	−0.37	−0.35	−0.24	−0.01	−0.05	−0.19
	*p*	0.001	0.002	0.040	0.030	0.001	0.001	0.030	0.410	0.680	0.090
Dry mouth SC ‡	r	0.28	0.31	0.15	0.16	−0.26	−0.19	0.20	−0.13	−0.17	0.02
	*p*	0.010	0.004	0.170	0.130	0.020	0.090	0.860	0.230	0.140	0.900

^☆^ Spearman’s rank correlation coefficient. † Oropharyngeal dysfunction cluster: mouth/throat sore, trismus, taste changes, difficulty swallowing, and hoarseness. ‡ Dry mouth cluster: dry mouth and sticky saliva.

**Table 6:** Stepwise regression analysis of the two clusters and clinical characteristics, quality of life coefficients (n = 83).

Oropharyngeal Dysfunction SC ^☆^					Dry Mouth SC †				
	B (95% Cl)	β §	t	*p*		B (95% Cl)	β §	t	*p*
Global quality of life	−0.017 (−0.11, −0.40)	−0.427	−4.380	<0.001	Global quality of life	−0.022 (−0.04, −0.01)	−0.268	−2.669	0.009
Treatment type ‡	2.406 (0.40, 4.41)	0.233	2.384	0.020	Treatment type ‡	1.765 (0.74, 2.80)	0.343	3.409	0.001
	R^2^ 0.239				R^2^ 0.191				
	Adjusted R^2^ 0.220				Adjusted R^2^ 0.171				

^☆^ Oropharyngeal dysfunction cluster: mouth/throat sore, trismus, taste changes, difficulty swallowing, and hoarseness: The independent variables included treatment type, anxiety, depression, social isolation, global QoL, physical function QoL, and role function QoL. † Dry mouth cluster: dry mouth and sticky saliva: The independent variables included treatment type, anxiety, depression, social isolation and global QoL. ‡ Dummy variables (1: Radiation therapy, 2: CRT). § Standardized partial regression coefficient.

## Data Availability

The datasets generated and/or analyzed during the current study are not publicly available due to privacy and ethical restrictions related to participant confidentiality.

## Supplementary Materials

The following supporting information can be downloaded at: https://www.mdpi.com/article/10.3390/nursrep16030103/s1, Table S1: Additional regression analyses including time since radiotherapy as a covariate.

## Public Involvement Statement

No public involvement in any aspect of this research.

## Guidelines and Standards Statement

This manuscript was drafted against the STROBE (Strengthening the Reporting of Observational Studies in Epidemiology) guidelines for observational crosssectional research.

## References

[1] Barsouk A., Aluru J.S., Rawla P., Saginala K. (2023). Epidemiology, risk factors, and prevention of head and neck squamous cell carcinoma. Med. Sci..

[2] Japan Society for Head and Neck Cancer Cancer Registry Committee (2020). Report of Head and Neck Cancer Registry of Japan: Clinical Statistics of Registered Patients. http://square.umin.ac.jp/jshnc/pdf/HNCreport_2020.pdf.

[3] Borras J.M., Barton M., Grau C., Corral J., Verhoeven R., Lemmens V., van Eycken L., Henke M., Gasparotto C., Majewski W. (2015). The impact of cancer incidence and stage on optimal utilization of radiotherapy: Methodology of a population-based analysis by the ESTRO-HERO project. Radiother. Oncol..

[4] Japanese Society for Radiation Oncology (2024). JASTARO Guidelines 2024 for Radiotherapy Treatment Planning.

[5] Iwanaga K., Ishibashi Y., Maki K., Sato Y., Saito Y., Yamamoto T., Shimizu T., Sakamoto T., Nakayama M., Yoshida R. (2023). Two-year evolution of quality of life following radiotherapy and/or chemotherapy in patients with head and neck cancer. Asia-Pac. J. Oncol. Nurs..

[6] Wang K., Tepper J. (2021). Radiation therapy-associated toxicity: Etiology, management, and prevention. CA Cancer J. Clin..

[7] Pinna R., Campus G., Cumbo E., Mura I. (2015). Xerostomia induced by radiotherapy: An overview of the physiopathology, clinical evidence, and management of the oral damage. Ther. Clin. Risk Manag..

[8] Strojan P., Hutcheson K.A., Eisbruch A., Beitler J.J., Langendijk J.A., Lee A.W.M., Corry J., Mendenhall W.M., Smee R., Rinaldo A. (2017). Treatment of late sequelae after radiotherapy for head and neck cancer. Cancer Treat. Rev..

[9] Raj R., Thankappan K., Janakiram C., Iyer S., Mathew A. (2020). Etiopathogenesis of trismus in patients with head and neck cancer: An exploratory literature review. Craniomaxillofac. Trauma Reconstr..

[10] Sardellitti L., Filigheddu E., Mastandrea G., Di Palma A., Milia E.P. (2025). Taste dysfunction in head and neck cancer: Pathophysiology and clinical management—A comprehensive review. Biomedicines.

[11] Larson A., Ma Y., Sun Z., Kingsbury J., Stepan K.O., Akthar A., Lorch J., Mittal B.B., Yadav P., Mierzwa M.L. (2025). Survivorship therapy needs after radiotherapy for head and neck cancer: Surveying opportunities for growth (STRONG). Support. Care Cancer.

[12] Kim H.-J., McGuire D.B., Tulman L., Barsevick A.M. (2005). Symptom clusters: Concept analysis and clinical implications for cancer nursing. Cancer Nurs..

[13] Kwekkeboom K.L. (2016). Cancer symptom cluster management. Semin. Oncol. Nurs..

[14] Mathew A., Tirkey A.J., Li H., Steffen A., Lockwood M.B., Patil C.L., Doorenbos A.Z. (2021). Symptom clusters in head and neck cancer: A systematic review and conceptual model. Semin. Oncol. Nurs..

[15] Genka T., Kamizato M. (2020). Housyasen ryouhougo no toukeibugan sabaiba ga kakaeru banki yugaizisyou no bunken kentou [Literature review on late toxicities Experienced by Head and Neck Cancer Survivors Following Radiation Therapy]. J. Okinawa Prefect. Coll. Nurs..

[16] Grove S.K., Burns N., Gray J.R., Kuroda Y., Itsumi I., Sato H. (2023). Burns & Grove’s Practice of Nursing Research, 9th ed.—Appraisal, Synthesis & Generation of Evidence.

[17] Maeda H. (2017). Trends in the treatment of head and neck malignancies in the Okinawa Prefecture. Ryukyu Med. J..

[18] Miyaji T., Iioka Y., Kuroda Y., Yamamoto D., Iwase S., Goto Y., Tsuboi M., Odagiri H., Tsubota Y., Kawaguchi T. (2017). Japanese translation and linguistic validation of the US National Cancer Institute’s PRO-CTCAE. J. Patient Rep. Outcomes.

[19] Aggarwal P., Zaveri J.S., Goepfert R.P., Shi Q., Du X.L., Swartz M., Lai S.Y., Fuller C.D., Lewin J.S., Piller L.B. (2019). Swallowing-Related Outcomes Associated with Late Lower Cranial Neuropathy in Long-Term Oropharyngeal Cancer Survivors: Cross-Sectional Survey Analysis. Head Neck.

[20] Nasri-Heir C., Zagury J.G., Thomas D., Ananthan S. (2015). Burning mouth syndrome: Current concepts. J. Indian Prosthodont. Soc..

[21] Hubler A., Cooper C., Heinzman K., Hapner E., Boswell W., McDonald A.M. (2024). Characterizing voice changes and primary aberrant features in survivors of locally advanced head and neck cancer treated with radiation therapy. Int. J. Radiat. Oncol. Biol. Phys..

[22] Theunissen E.A.R., Bosma S.C.J., Zuur C.L., Spijker R., van der Baan S., Dreschler W.A., de Boer J.P., Balm A.J.M., Rasch C.R.N. (2014). Sensorineural hearing loss in patients with head and neck cancer after chemoradiotherapy and radiotherapy: A systematic review of the literature. Head Neck.

[23] Pedroso C.M., Migliorati C.A., Epstein J.B., Ribeiro A.C.P., Brandão T.B., Lopes M.A., de Goes M.F., Santos-Silva A.R. (2022). Over 300 Radiation Caries Papers: Reflections From the Rearview Mirror. Front. Oral Health.

[24] Basch E., Reeve B.B., Mitchell S.A., Clauser S.B., Minasian L.M., Dueck A.C., Mendoza T.R., Hay J., Atkinson T.M., Abernethy A.P. (2014). Development of the National Cancer Institute’s Patient-Reported Outcomes Version of the Common Terminology Criteria for Adverse Events (PRO-CTCAE). J. Natl. Cancer Inst..

[25] Ho K.F., Farnell D.J.J., Routledge J.A., Burns M.P., Sykes A.J., Slevin N.J., Davidson S.E. (2009). Developing a CTCAE’s Patient Questionnaire for Late Toxicity after Head and Neck Radiotherapy. Eur. J. Cancer.

[26] Toth G., Tsukuda M. (2004). The European Organisation for Research and Treatment of Cancer (EORTC) Quality of Life Questionnaire for Japanese Patients with Head and Neck Cancer—The Japanese Version of QLQ-H&N35. Jpn. J. Cancer Chemother..

[27] Zigmond A.S., Snaith R.P. (1993). Hospital Anxiety and Depression Scale (HADS scale). Arch. Psychiatr. Diagn. Clin. Eval..

[28] Zigmond A.S., Snaith R.P. (1983). The Hospital Anxiety and Depression Scale. Acta Psychiatr. Scand..

[29] Singer S., Kuhnt S., Götze H., Hauss J., Hinz A., Liebmann A., Krauß O., Lehmann A., Schwarz R. (2009). Hospital anxiety and depression scale cutoff scores for cancer patients in acute care. Br. J. Cancer..

[30] Asakura S., Inoue S., Sasaki H., Sasaki Y., Kitagawa N., Inoue T., Denda K., Ito M., Matsubara R., Koyama T. (2002). Reliability and validity of Liebowitz Social Anxiety Scale (LSAS) Japanese version. Clin. Psychiatry.

[31] Liebowitz M.R. (1987). Social phobia. Mod. Probl. Pharmacopsychiatry.

[32] Masuda Y., Tadaka E., Dai Y. (2012). Reliability and Validity of the Japanese Version of the UCLA Loneliness Scale Version 3 among the Older Population. J. Jpn. Acad. Com. Health Nurs..

[33] Russell D., Peplau L.A., Cutrona C.E. (1980). The revised UCLA Loneliness Scale: Concurrent and discriminant validity evidence. J. Pers. Soc. Psychol..

[34] Toth G., Sakaguchi T., Mikami Y., Hirose H., Tsukuda M. (2005). A pilot study of the translation, cultural adaptation and validation of the EORTC head and neck cancer quality of life questionnaire module (QLQ-H&N35) for use in Japan. Auris Nasus Larynx.

[35] Aaronson N.K., Ahmedzai S., Bergman B., Bullinger M., Cull A., Duez N.J., Filiberti A., Flechtner H., Fleishman S.B., de Haes J.C.J.M. (1993). The European organization for research and treatment of cancer QLQ-C30: A quality-of-life instrument for use in international clinical trials in oncology. J. Natl. Cancer Inst..

[36] Russell J., Wong M.L., Mackin L., Paul S.M., Cooper B.A., Hammer M., Conley Y.P., Wright F., Levine J.D., Miaskowski C. (2019). Stability of Symptom Clusters in Patients with Lung Cancer Receiving Chemotherapy. J. Pain Symptom Manag..

[37] Skerman H.M., Yates P.M., Battistutta D. (2012). Identification of Cancer-Related Symptom Clusters: An Empirical Comparison of Exploratory Factor Analysis Methods. J. Pain Symptom Manag..

[38] Palmieri M., Sarmento D.J.S., Falcão A.P., Cruz M.H.S. (2021). Frequency and Evolution of Acute Oral Complications in Patients Undergoing Radiochemotherapy Treatment for Head and Neck Squamous Cell Carcinoma. Ear Nose Throat J..

[39] Muehlebach M.E., Pradeep S., Chen X., Arnold L., Arthur A.E., Gan G.N., Thomas S.M. (2025). Radiation-Induced Fibrosis (RIF) in Head and Neck Squamous Cell Carcinoma (HNSCC): A Review. Cells.

[40] Haidar Z.S. (2022). Radiation-Induced Salivary Gland Damage/Dysfunction in Head and Neck Cancer: Nano-Bioengineering Strategies and Artificial Intelligence for Prevention, Therapy and Reparation. J. Radiol. Oncol..

[41] Jiang N., Zhao Y., Jansson H., Chen X., Mårtensson J. (2018). Experiences of Xerostomia after Radiotherapy in Patients with Head and Neck Cancer: A Qualitative Study. J. Clin. Nurs..

[42] Goh E.Z., Johnson N.R., Batstone M., Beech N. (2023). The Dental Management of Patients Irradiated for Head and Neck Cancer. Br. Dent. J..

[43] Castro K.M., Tonorezos E.S. (2023). Introduction to the Special Section: Treatment-Related Hearing Loss and Its Impact on Cancer Survivors. J. Cancer Surviv..

[44] Yu Z., Xu C., Song B., Zhang S., Chen C., Li C., Zhang S. (2023). Tissue Fibrosis Induced by Radiotherapy: Current Understanding of the Molecular Mechanisms, Diagnosis and Therapeutic Advances. J. Transl. Med..

[45] Albers J.W., Chaudhry V., Cavaletti G., Donehower R.C. (2014). Interventions for Preventing Neuropathy Caused by Cisplatin and Related Compounds. Cochrane Database Syst. Rev..

[46] Paz C., Glassey A., Frick A., Sattar S., Zaorsky N.G., Blitzer G.C., Kimple R.J. (2024). Cancer Therapy–Related Salivary Dysfunction. J. Clin. Investig..

[47] Jensen S.B., Vissink A., Limesand K.H., Reyland M.E. (2019). Salivary Gland Hypofunction and Xerostomia in Head and Neck Radiation Patients. J. Natl. Cancer Inst. Monogr..

[48] Rogers S.N., Simo R. (2016). Follow-Up and Survivorship in Head and Neck Cancer. Clin. Oncol. (R. Coll. Radiol.).

[49] Starmer H.M., McCarroll L., Goldsmith T., Kizner J., Hamilton J., Yao T., Beadle B.M. (2025). One-year swallowing outcomes in a head and neck cancer cohort: The impact of adherence to swallowing exercises and feeding tube use. Dysphagia.

