# Supplementary material for: Symptom Clusters and Related Factors of Late Toxicities in Head and Neck Cancer Survivors After Radiation Therapy: A Cross-Sectional Study
Source: Nurs Rep. 2026 Mar 23;16(3):103. doi: 10.3390/nursrep16030103 (PMC13029374; doi:10.3390/nursrep16030103)
Supplement: Supplementary file 1 [file nursrep-16-00103-s001.zip › nursrep-4161784-supplementary.pdf]

Supplementary Table S1. Additional regression analyses including time since radiotherapy as a covariate (n = 83)

Oropharyngeal dysfunction SC☆					Dry mouth SC†				
	B (95%Cl)	β§	t	p		B (95%Cl)	β§	t	p
Global quality of life	-.017 (-0.11, -0.40)	-0.427	-4.380	＜0.001	Global quality of life	-0.022 (-0.04, -0.01)	-0.268	-2.669	0.009
Treatment type‡	2.406 (0.40, 4.41)	0.233	2.384	0.020	Treatment type‡	1.765 (0.74, 2.80)	0.343	3.409	0.001
			R^2	0.239			R^2		0.191
			Adjusted R^2	0.220				Adjusted R^2	0.171

☆Oropharyngeal dysfunction cluster: mouth/throat sore, trismus, taste changes, difficulty swallowing, and hoarseness: The independent variables included treatment type, anxiety, depression, social isolation, global QoL, physical function QoL, role function QoL, and **time since radiotherapy**.

†Dry mouth cluster: dry mouth and sticky saliva: The independent variables included treatment type, anxiety, depression, social isolation global QoL, and **time since radiotherapy.**

‡ Dummy variables (1: Radiation therapy, 2: CRT)

§ Standardized partial regression coefficient

Note: Time since radiotherapy was added as a candidate independent variable in the additional stepwise regression analyses. Inclusion of this variable did not materially alter the main findings.
